# Renal function and urinary tubular biomarker abnormalities in untreated and entecavir- or tenofovir disoproxil fumarate–treated patients with chronic hepatitis B: a retrospective cross-sectional study

**DOI:** 10.3389/fmed.2026.1928760

**Published:** 2026-08-26

**Authors:** Haiyan Lan, Qilong Nie, Qiuyan Liang, Caiyang Huang, Jian Ren, Wenya Peng, Ronghuo Zhu, Jianhong Li

**Affiliations:** 1 The Eighth Clinical Medical College, Guangzhou University of Chinese Medicine, Foshan, Guangdong, China; 2 Foshan Hospital of Traditional Chinese Medicine, Guangzhou University of Chinese Medicine, Foshan, Guangdong, China

**Keywords:** chronic hepatitis B, entecavir, estimated glomerular filtration rate, renal tubular biomarkers, tenofovir disoproxil fumarate, *α*1-Microglobulin, *β*2-microglobulin

## Abstract

**Background:**

Renal safety is an important consideration during nucleos(t)ide analogue (NA) therapy for chronic hepatitis B (CHB). Tenofovir disoproxil fumarate (TDF) may affect proximal tubular function, while serum creatinine and estimated glomerular filtration rate (eGFR) primarily reflect glomerular filtration. This study compared glomerular-function measures and urinary tubular biomarker patterns among NA-untreated, entecavir (ETV)-treated, and TDF-treated patients with CHB.

**Methods:**

This single-center retrospective cross-sectional study included 242 adults with CHB assessed between June 2025 and June 2026: 113 NA-untreated, 64 ETV-treated, and 65 TDF-treated participants. For treated participants, the single index assessment was obtained after at least 12 months of continuous corresponding monotherapy. Serum creatinine, eGFR, urinary *α*1-microglobulin (A1M), urinary *β*2-microglobulin (B2M), and urinary microalbumin (U-mAlb) were compared among groups. eGFR was calculated using the 2009 CKD-EPI creatinine equation and analyzed primarily as a continuous measure; eGFR <90 mL/min/1.73 m^2^ was retained as a secondary screening outcome. Urinary biomarkers were measured as spot-urine concentrations without urinary creatinine normalization.

**Results:**

Urinary A1M ≥ 12.0 mg/L occurred in 13.3%, 42.2%, and 67.7% of NA-untreated, ETV-treated, and TDF-treated participants, respectively; corresponding frequencies of urinary B2M > 0.30 mg/L were 25.7%, 48.4%, and 75.4%. Both A1M and B2M concentration-threshold exceedance were more frequent in TDF-treated than ETV-treated participants (Bonferroni-adjusted *P* = 0.0108 and *P* = 0.0048, respectively), and continuous B2M was also higher with TDF (adjusted *P* = 0.0074). Median eGFR was 112.98 (102.47–119.36), 104.79 (92.08–112.39), and 104.47 (91.24–116.58) mL/min/1.73 m^2^, respectively (overall *P* = 0.0008). Both treated groups had lower continuous eGFR than the NA-untreated group, whereas serum creatinine, continuous eGFR, and the frequency of eGFR <90 mL/min/1.73 m^2^ did not differ between ETV and TDF (all Bonferroni-adjusted *P* = 1.0000).

**Conclusion:**

TDF-treated participants showed a more prominent urinary A1M/B2M concentration-based biomarker pattern than ETV-treated participants, whereas conventional glomerular-function measures did not differ between the two treated groups. These findings represent cross-sectional associations rather than evidence of treatment-induced renal injury. The absence of pretreatment and serial renal measurements, urinary creatinine normalization, and a complete proximal tubular dysfunction assessment requires cautious interpretation and prospective longitudinal confirmation.

## 1 Introduction

Chronic hepatitis B (CHB) is a chronic liver disease caused by persistent infection with hepatitis B virus (HBV) and is generally diagnosed by hepatitis B surface antigen (HBsAg) positivity lasting for more than 6 months (1). Patients with CHB are often asymptomatic during the early stages of infection; however, with disease progression, they may gradually develop hepatic inflammation, fibrosis, cirrhosis, and hepatocellular carcinoma (2). Although universal hepatitis B vaccination and prevention of mother-to-child transmission have substantially reduced the risk of new HBV infections, CHB remains a major public health burden in China. A recent epidemiological report showed that the prevalence of HBsAg in the general Chinese population was 5.86% in 2020, with approximately 75 million individuals living with chronic HBV infection, accounting for nearly one-third of the global HBV-infected population. In addition, approximately 1.05 million cases of hepatitis B were reported in China in 2023 (3).

Long-term suppression of HBV replication remains a cornerstone of clinical management for preventing or delaying disease progression in patients with CHB (1). Nucleos(t)ide analogues (NAs) exert potent antiviral effects by inhibiting HBV polymerase/reverse transcriptase activity and thereby suppressing viral DNA synthesis (1). Entecavir (ETV), tenofovir disoproxil fumarate (TDF), and tenofovir alafenamide (TAF) are potent NAs with a high genetic barrier to resistance and are recommended as preferred oral antiviral agents for CHB (1). Long-term NA therapy can achieve sustained viral suppression, improve hepatic necroinflammation and fibrosis, and reduce the risks of cirrhosis and hepatocellular carcinoma (1, 2). However, because prolonged or lifelong treatment is frequently required, renal safety remains an important consideration during therapy, particularly for patients receiving TDF or those with pre-existing renal risk factors (4, 5).

TDF has received particular attention because tenofovir is taken up by proximal tubular cells, and excessive intracellular exposure may lead to tubular dysfunction (4, 5). Clinically, TDF-associated renal abnormalities may manifest as a reduction in estimated glomerular filtration rate (eGFR), low-molecular-weight proteinuria, hypophosphatemia, glycosuria, or impaired tubular reabsorption (4, 5). By contrast, ETV has generally demonstrated a favorable renal safety profile, although renal function monitoring remains necessary, particularly in older patients and those with pre-existing renal impairment or metabolic comorbidities (6, 7). Comparative evidence between ETV and TDF has not been entirely consistent. A systematic review and meta-analysis found that TDF was associated with statistically greater increases in serum creatinine and reductions in eGFR than ETV during 6–24 months of treatment, although the absolute between-group differences were generally small and the certainty of the available evidence was limited (6). Similarly, the multinational REAL-B study reported that TDF was associated with a greater long-term risk of renal function decline than ETV, emphasizing the need to consider baseline renal function, age, treatment duration, and comorbidity burden when selecting and monitoring antiviral therapy (7).

TAF was developed to deliver tenofovir more efficiently to hepatocytes while reducing systemic tenofovir exposure (8). Randomized phase III studies demonstrated that TAF achieved antiviral efficacy comparable to that of TDF while producing more favorable changes in renal and bone safety parameters (8). A subsequent network meta-analysis comparing ETV, TDF, and TAF also suggested that long-term TDF treatment had a less favorable renal and bone safety profile than ETV and TAF (9). Nevertheless, differences among these agents may vary according to the study population, baseline renal reserve, duration of treatment, and renal outcomes evaluated. Therefore, the available evidence should not be interpreted as indicating that clinically significant nephrotoxicity occurs uniformly in all patients receiving TDF (6–9).

Recent studies have also compared the renal safety of TAF and ETV. A nationwide South Korean cohort study of treatment-naïve patients with CHB found no significant difference between TAF and ETV in the incidence of chronic kidney disease or end-stage renal disease after propensity score matching (10). In addition, Döngelli et al. compared TAF and ETV in liver transplant recipients during a 48-month retrospective follow-up and reported broadly comparable changes in renal function between the two treatment groups (11). However, the relatively small sample size, retrospective design, transplant setting, and potential effects of concomitant immunosuppressive therapy limit the direct generalizability of the latter findings to non-transplant patients with CHB. Collectively, contemporary evidence suggests that TAF and ETV generally demonstrate favorable renal safety profiles, whereas closer renal monitoring may be warranted during long-term TDF treatment, particularly in patients with additional renal risk factors (6–11).

In routine clinical follow-up, serum creatinine and eGFR are the most commonly used indicators for assessing renal function (12). Nevertheless, serum creatinine primarily reflects glomerular filtration and may remain within the laboratory reference range during the early stages of proximal tubular dysfunction (12, 13). Urinary *α*1-microglobulin (A1M) and *β*2-microglobulin (B2M) are low-molecular-weight proteins that are filtered by the glomeruli and subsequently reabsorbed and metabolized by proximal tubular cells. Impaired proximal tubular reabsorption may therefore lead to increased urinary A1M and B2M concentrations, making these biomarkers potentially useful for detecting tubular abnormalities (14, 15). Urinary albumin can provide complementary information regarding renal involvement (12). Accordingly, reliance on serum creatinine alone may fail to identify some early renal abnormalities occurring during long-term NA therapy (13). For untimed spot-urine samples, however, albumin concentration varies with urine dilution; standardized clinical albuminuria assessment is preferably based on a creatinine-normalized measure such as the albumin-to-creatinine ratio (ACR) rather than albumin concentration alone (12).

The assessment of renal function across different antiviral treatment groups is also affected by patient characteristics and treatment-selection factors. Aging is associated with a gradual decline in glomerular filtration function (16), Accordingly, the 2009 CKD-EPI creatinine equation used in this study incorporates serum creatinine, age, and sex as input variables (17). Moreover, hypertension, diabetes mellitus, hyperuricemia, fatty liver disease, and other comorbidities may be unevenly distributed among treatment groups and should therefore be considered as potential confounders in observational comparisons. Because treatment allocation in the present study was not randomized, the observed differences in renal outcomes may also partly reflect confounding by indication, physician prescribing preferences, and differences in baseline renal reserve.

Several previous studies have evaluated renal tubular abnormalities in patients with CHB receiving long-term NA therapy. Gara et al. reported that renal tubular dysfunction developed in approximately 15% of patients treated with adefovir or tenofovir for 2–9 years, suggesting that tubular abnormalities may emerge during prolonged nucleotide analogue exposure even when severe glomerular dysfunction is uncommon (18). Ji et al. retrospectively evaluated 69 outpatients with CHB receiving long-term NA therapy and found that early renal injury indicators were more sensitive than conventional renal indices for identifying mild renal abnormalities (13). In addition, Atef et al. specifically investigated urinary *β*2-microglobulin as a potential marker of TDF-related tubular dysfunction in patients with CHB, further supporting the potential value of low-molecular-weight urinary proteins in renal monitoring (19). However, these studies generally involved selected treated populations, focused on individual antiviral regimens or limited biomarker panels, and did not simultaneously compare NA-untreated, ETV-treated, and TDF-treated patients using eGFR together with urinary A1M, B2M, and U-mAlb.

Taken together, current evidence indicates that renal findings during NA therapy may differ according to the renal domain assessed, with conventional glomerular measures and urinary low-molecular-weight proteins providing non-equivalent information (6–11, 13, 18, 19). Comparatively few real-world studies have simultaneously evaluated these domains across NA-untreated patients and patients receiving ETV or TDF. We therefore hypothesized that the TDF-treated group would show a more prominent urinary A1M/B2M concentration pattern than the other exposure groups, without assuming that such a pattern would necessarily be accompanied by worse serum creatinine or eGFR. Accordingly, the primary descriptive objective was to compare urinary tubular biomarker patterns alongside glomerular-function measures among NA-untreated, ETV-treated, and TDF-treated patients with CHB. The categorical eGFR <90 mL/min/1.73 m^2^ analysis was retained only as a secondary exploratory screening analysis. Throughout the manuscript, differences observed among antiviral-exposure groups are therefore interpreted as cross-sectional associations or biomarker patterns and not as evidence of treatment-induced renal injury. The intended contribution is therefore comparative rather than mechanistic: to characterize, within a single real-world CHB cohort, urinary tubular biomarker patterns and glomerular-function measures across NA-untreated, ETV-treated, and TDF-treated patients.

## 2 Methods

### 2.1 Study design and participants

This single-center retrospective cross-sectional study was conducted at Foshan Hospital of Traditional Chinese Medicine. Adult patients with CHB who were admitted to the hospital or followed up in the outpatient clinic between June 2025 and June 2026 were screened.

CHB was diagnosed according to the national Guidelines for the Prevention and Treatment of Chronic Hepatitis B and was consistent with the diagnostic definitions provided in the World Health Organization guidelines and the European Association for the Study of the Liver Clinical Practice Guidelines (20, 21). CHB was defined primarily by documented hepatitis B surface antigen positivity for at least 6 months. Virological, biochemical, imaging, clinical, and, when available, histopathological findings were used for further disease characterization. For patients already receiving antiviral therapy, the diagnosis was confirmed using initial diagnostic records or pretreatment medical documentation.

Eligible patients were aged ≥18 years, had traceable medical records, and had available demographic, antiviral treatment, liver function, renal function, and urinary biomarker data. Patients in the entecavir (ETV) and tenofovir disoproxil fumarate (TDF) groups were required to have received continuous corresponding monotherapy for at least 12 months before the index laboratory assessment. Patients in the nucleos(t)ide analogue (NA)-untreated group had no documented history of treatment with ETV, TDF, tenofovir alafenamide, lamivudine, adefovir, telbivudine, or other anti-HBV NAs before the index assessment. The ≥12-month criterion served only as an eligibility threshold; exact treatment duration beyond this threshold and cumulative antiviral exposure were not consistently available and were not analyzed. Exposure classification was based on the documented antiviral agent rather than quantified dose intensity. Participant-level antiviral dose, renal dose-adjustment history, and formal adherence measures were not available as analyzable variables in the retrospective dataset and therefore were not incorporated into the exposure definition or statistical models. The requirement for continuous monotherapy refers to documented treatment with the same antiviral agent and should not be interpreted as direct verification of day-to-day adherence or unchanged dosing throughout the exposure period.

Patients were excluded if they had decompensated cirrhosis, hepatocellular carcinoma, hepatorenal syndrome, other severe liver-related complications, previously diagnosed primary renal disease or chronic kidney disease, coinfection with another hepatitis virus, or another active liver disease of a clearly defined etiology. Additional exclusion criteria included antiviral treatment for <12 months in the ETV or TDF group; switching, combining, or changing anti-HBV agents during the observation period; recent exposure to potentially nephrotoxic medications; coexisting malignancy, severe infection, or severe cardiovascular or cerebrovascular disease; pregnancy or lactation; and incomplete key clinical or laboratory data. These criteria were selected because the corresponding conditions or exposures could independently affect renal function, interfere with accurate classification of antiviral exposure, or compromise the reliability of renal outcome assessment. Compensated cirrhosis and imaging-detected fatty liver disease were retained as documented comorbidities unless they were considered the primary cause of liver disease.

At the index laboratory assessment, participants were classified into the NA-untreated, ETV-treated, or TDF-treated group. For treated patients, the index assessment was an eligible laboratory evaluation performed after at least 12 months of continuous corresponding monotherapy. For untreated patients, the first eligible assessment during the study period was used. Renal outcomes were evaluated at this single index assessment. Because pretreatment renal parameters and repeated within-person renal measurements were unavailable for all participants, the study assessed cross-sectional differences among the three exposure groups rather than longitudinal changes or treatment-induced renal decline. The ≥12-month treatment criterion was used only to define antiviral exposure status and did not permit assessment of treatment-emergent renal change. Exact treatment duration beyond the eligibility threshold and cumulative exposure could not be quantified; therefore, patients with potentially different lengths of therapy were analyzed within the same exposure category, and no exposure-duration analysis was performed. Accordingly, the ETV- and TDF-treated categories represent agent-based exposure groups rather than standardized dose- or adherence-defined exposure levels.

**Figure 1:**
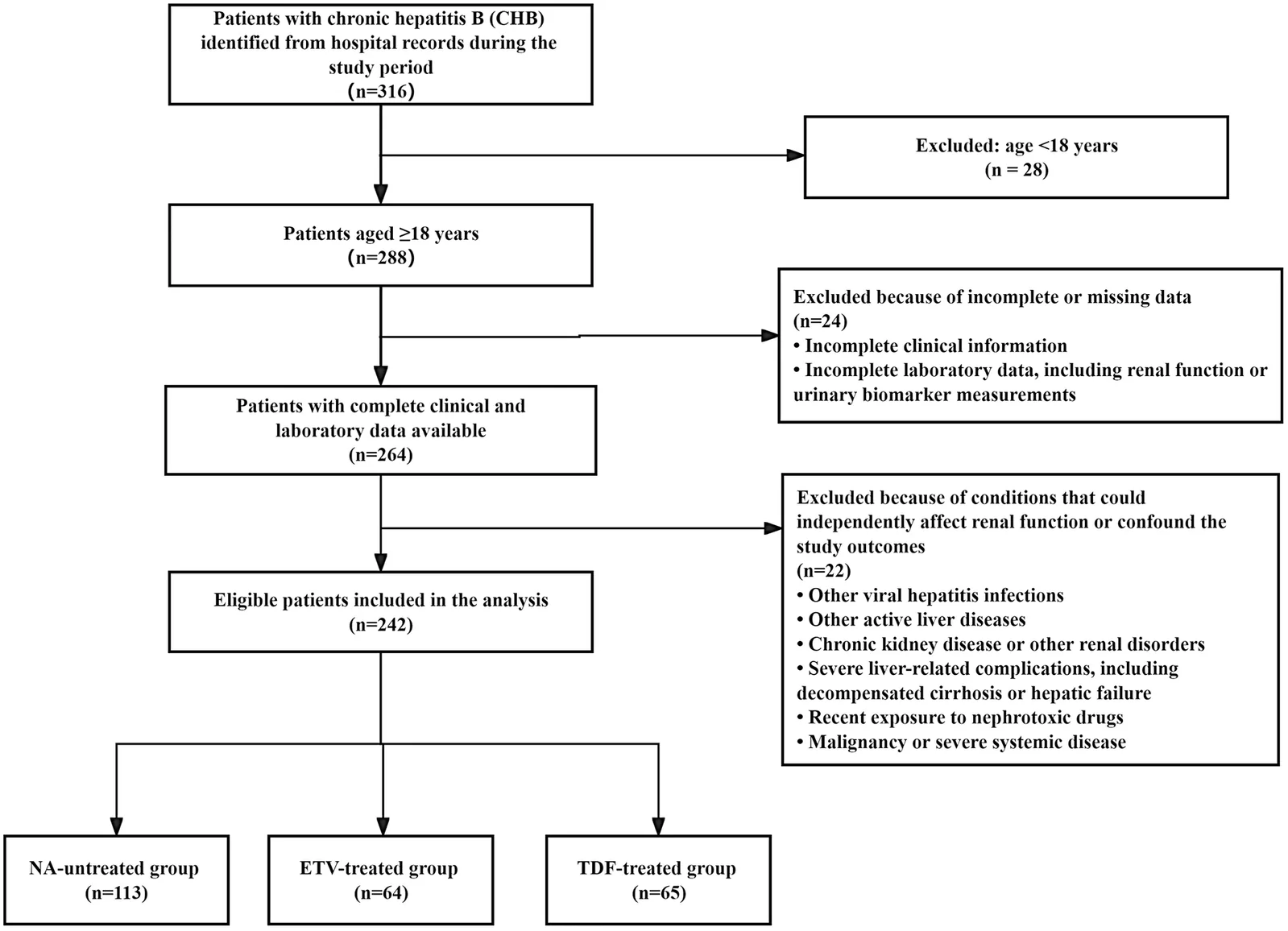
Flow diagram of participant selection and classification by antiviral exposure group. A total of 316 patient records were screened. After excluding 28 patients aged <18 years, 24 patients with incomplete or missing data, and 22 patients with conditions that could independently affect renal function or confound the study outcomes, 242 eligible participants were included in the analysis. These participants were classified into the NA-untreated group (*n* = 113), ETV-treated group (*n* = 64), and TDF-treated group (*n* = 65). CHB, chronic hepatitis B; ETV, entecavir; NA, nucleos(t)ide analogue; TDF, tenofovir disoproxil fumarate.

The participant selection process is summarized in Figure 1. Of the 316 patient records screened, 242 participants met the eligibility criteria and were included in the final analysis.

### 2.2 Data collection and outcome definitions

Demographic, clinical, and laboratory data were retrospectively extracted from the hospital electronic medical record system. Demographic variables included age, sex, and body weight. Clinical variables included antiviral exposure status and the following documented comorbidities recorded separately: hypertension, diabetes mellitus, hyperuricemia, fatty liver disease, and compensated cirrhosis. These conditions were treated as distinct clinical variables and were not combined into a single composite comorbidity covariate in the final inferential models. Individual comorbidities were not mutually exclusive because some participants had more than one condition. The medical records established the documented presence of these conditions but did not provide sufficiently complete and standardized information on their severity, duration, treatment status, or degree of control for comparative analysis. In particular, longitudinal blood-pressure measurements, HbA1c, duration of hypertension or diabetes, and sufficiently detailed information to classify diabetic kidney disease were not available as analyzable variables.

Laboratory variables included hepatitis B virus DNA status, hepatitis B surface antigen, alanine aminotransferase (ALT), uric acid (UA), serum creatinine, estimated glomerular filtration rate (eGFR), urinary *α*1-microglobulin (A1M), urinary *β*2-microglobulin (B2M), and urinary microalbumin (U-mAlb). HBV DNA at the index assessment was categorized as <100 or ≥100 IU/mL for descriptive comparisons. For treated participants, this measurement was obtained after antiviral exposure and may therefore reflect treatment response or current virological status rather than pretreatment disease status. eGFR was calculated from age, sex, and serum creatinine using the 2009 CKD-EPI creatinine equation, which was the standard equation adopted by the institutional laboratory during the study period (17): eGFR = 141 × min(Scr/*κ*, 1)*^α^* × max(Scr/*κ*, 1)^−1.209^ × 0.993^Age^ × 1.018 (if female), where Scr is serum creatinine in mg/dL, *κ* is 0.7 for women and 0.9 for men, and *α* is −0.329 for women and −0.411 for men. Serum creatinine values recorded in μmol/L were converted to mg/dL by dividing by 88.4 before calculation. No Black race coefficient was applied. The 2021 race-free CKD-EPI equation was not used. Urinary A1M, B2M, and U-mAlb were extracted as spot-urine concentrations reported in mg/L. Paired urinary creatinine measurements were not available in the retrospective dataset; therefore, A1M/creatinine, B2M/creatinine, and albumin-to-creatinine ratios could not be calculated. Serum phosphate, urinary phosphate excretion or fractional phosphate wasting, glycosuria, serum bicarbonate, and other components of a formal proximal tubular dysfunction assessment were not systematically available in the retrospective dataset and were not used to diagnose Fanconi syndrome or drug-induced proximal tubular toxicity.

Because this study relied on retrospectively extracted laboratory results, participant-level pre-analytical information for urinary B2M—including urine pH, any pH adjustment or preservative use, the exact interval from specimen collection to analysis, storage temperature and duration, and sample-stability documentation—was not available for analysis. Urinary B2M is susceptible to degradation in acidic urine, which may lower measured concentrations and complicate interpretation of spot-urine B2M values (22). Accordingly, B2M findings were interpreted as concentration-based exploratory biomarker observations rather than as definitive evidence of proximal tubular injury.

Laboratory reference thresholds used for categorical analyses were as follows: ALT >50 U/L, UA >428 μmol/L, serum creatinine >97 μmol/L, U-mAlb ≥15.0 mg/L, A1M ≥ 12.0 mg/L, and B2M > 0.30 mg/L. The urinary thresholds represent concentration-based institutional reference limits rather than creatinine-normalized classifications; in particular, U-mAlb ≥15.0 mg/L was not considered a standardized albuminuria category.

Renal function was evaluated primarily using eGFR as a continuous variable, thereby retaining the full information contained in the observed eGFR distribution. An eGFR <90 mL/min/1.73 m^2^ was additionally examined as a secondary categorical screening outcome. A single value below this threshold was not considered equivalent to a clinical diagnosis of chronic kidney disease. Urinary A1M ≥12.0 mg/L, urinary B2M >0.30 mg/L, and U-mAlb ≥15.0 mg/L were evaluated as concentration-threshold urinary biomarker outcomes. The U-mAlb threshold was not considered equivalent to a standardized clinical albuminuria category because paired urinary creatinine and ACR were unavailable. A stricter threshold of eGFR <60 mL/min/1.73 m^2^ was examined descriptively in sensitivity analysis. Accordingly, the urinary biomarker analyses should be interpreted as comparisons of spot-urine concentrations and concentration-based threshold exceedance, not as standardized creatinine-normalized excretion measures.

### 2.3 Statistical analysis

The normality of continuous variables was assessed using the Shapiro–Wilk test. Continuous variables that were approximately normally distributed were presented as mean ± standard deviation and compared among the three antiviral exposure groups using one-way analysis of variance. Variables that did not meet the normality assumption were presented as median (interquartile range) and compared using the Kruskal–Wallis test. Categorical variables were presented as *n* (%) and compared using the Pearson chi-square test or the Fisher–Freeman–Halton exact test, as appropriate. For eGFR, the continuous-variable comparison was treated as the principal analysis of glomerular filtration across exposure groups; the threshold-based eGFR analyses were secondary and exploratory.

For variables with a statistically significant overall comparison across the three groups, *post hoc* pairwise comparisons were subsequently performed. Continuous variables were compared using pairwise Mann–Whitney *U* tests. Categorical variables were compared using pairwise Pearson chi-square tests or Fisher's exact tests, as appropriate. *P* values from the three pairwise comparisons—NA-untreated vs. ETV-treated, NA-untreated vs. TDF-treated, and ETV-treated vs. TDF-treated—were adjusted using the Bonferroni method. A Bonferroni-adjusted *P* value <0.05 was considered statistically significant. These overall and pairwise comparisons were descriptive, unadjusted comparisons of characteristics observed at the index assessment and were not interpreted as estimates of causal treatment effects.

Exploratory associations among HBsAg, ALT, uric acid, serum creatinine, eGFR, urinary A1M, urinary B2M, and U-mAlb were evaluated separately within the NA-untreated, ETV-treated, and TDF-treated groups using Spearman rank correlation coefficients. Correlation matrices were visualized using heatmaps. These analyses were interpreted descriptively, and no formal statistical comparisons of correlation coefficients were performed across the three exposure groups. Correlations involving serum creatinine and eGFR were retained for descriptive completeness, but because serum creatinine is a direct input to the eGFR equation, the creatinine–eGFR correlation was considered mathematically expected and was not interpreted as an independent biological association.

As a secondary exploratory analysis, binary logistic regression was performed for the categorical screening outcome of eGFR <90 mL/min/1.73 m^2^. Antiviral exposure group was included as the principal explanatory variable, with the NA-untreated group serving as the reference category. Model 1 was unadjusted. Because only 35 participants met the categorical outcome, the adjusted analysis was deliberately restricted to a parsimonious Model 2 containing antiviral exposure group, age, and sex. We did not use an “any documented comorbidity” composite in the final regression because hypertension, diabetes mellitus, hyperuricemia, fatty liver disease, and compensated cirrhosis are clinically heterogeneous and a single binary indicator would not provide robust confounding control. Conversely, entering all five conditions separately was not considered statistically reliable because several were infrequent and the limited event count would markedly increase overfitting and unstable estimation. Urinary A1M, B2M, and U-mAlb were not included as covariates because they were evaluated as renal biomarker outcomes rather than covariates for confounding control. Odds ratios and corresponding 95% confidence intervals were reported. HBV DNA category and ALT measured at the index assessment were not entered as additional confounders solely because they differed among groups, because in treated participants these variables were measured after antiviral exposure and may partly reflect treatment response or current disease activity rather than pretreatment characteristics. Accordingly, Model 2 was interpreted as providing only partial confounding control rather than a causal drug-effect estimate. Age and sex were retained solely as demographic adjustment covariates to account for measured group differences; because both are components of the 2009 CKD-EPI creatinine equation used to calculate eGFR, their coefficients were not interpreted as independent biological effects. Given the small number of events, the inability to adjust robustly for the individual comorbidities, and the wide confidence intervals, all regression estimates were regarded as imprecise and exploratory. More specifically, the marked treated-vs.-untreated difference in HBV DNA was not statistically “balanced” by adjustment for the index HBV DNA category, because in the treated groups this variable was measured after ≥12 months of antiviral exposure and is potentially treatment-responsive. The three exposure groups were retained to address the original descriptive objective of comparing renal and urinary biomarker patterns across real-world antiviral-exposure states, not to emulate a randomized treated-vs.-untreated contrast.

Two sensitivity analyses were retained. First, a stricter renal threshold of eGFR <60 mL/min/1.73 m^2^ was examined. Because only three participants met this criterion, adjusted logistic regression was not performed and the results were reported descriptively. Second, an exploratory analysis restricted to ETV- and TDF-treated participants was retained to directly compare TDF with ETV for the eGFR <90 mL/min/1.73 m^2^ screening outcome after adjustment for age, sex, body weight, and the presence of any documented comorbidity. Because the composite comorbidity variable combines clinically heterogeneous conditions and the number of outcome events in the treated subgroup was limited, this treated-only model is interpreted cautiously as a supportive sensitivity analysis rather than as robust confounding control. The previously reported analysis excluding all participants with documented comorbidities was removed because the further reduction in sample size and events produced very imprecise estimates.

The demographic, antiviral exposure, renal function, urinary biomarker, and individual documented-comorbidity variables required for the descriptive analyses were available for all 242 participants. No missing values were imputed, and all 242 eligible participants were included in the primary analyses.

All statistical tests were two-sided. An overall *P* value <0.05 was considered statistically significant, and Bonferroni-adjusted *P* values were used to determine statistical significance in *post hoc* pairwise comparisons. Statistical analyses were performed using IBM SPSS Statistics, version 27.0 (IBM Corp., Armonk, NY, USA).

### 2.4 Ethical considerations

The study protocol was approved by the Medical Ethics Committee of Foshan Hospital of Traditional Chinese Medicine (approval No. KY(2024)311; initial approval date: October 12, 2024). Written informed consent for participation and the use of clinical data was obtained from all participants. All data were de-identified before analysis, and patient confidentiality was strictly protected. The study was conducted in accordance with the principles of the Declaration of Helsinki and relevant institutional ethical requirements.

## 3 Results

### 3.1 Demographic, clinical, virological, and laboratory characteristics

A total of 242 participants were included: 113 in the NA-untreated group, 64 in the ETV-treated group, and 65 in the TDF-treated group. Overall comparisons showed significant differences among the three groups in age, sex distribution, HBV DNA category, ALT, serum creatinine, HBsAg, urinary A1M, urinary B2M, U-mAlb, eGFR, and the prevalence of fatty liver disease. In contrast, body weight, uric acid, hypertension, diabetes mellitus, hyperuricemia, and compensated cirrhosis did not differ significantly among the groups (Table 1). These variables characterize the groups at the index assessment and should not be interpreted as pretreatment baseline characteristics.

**Table 1:** Demographic, clinical, virological, and laboratory characteristics by antiviral exposure group.

Variable and data presentation	NA-untreated (*n* = 113)	ETV-treated (*n* = 64)	TDF-treated (*n* = 65)	Overall *P* value
Age, years, median (IQR)	40.00 (34.00–46.00)	47.50 (39.00–53.00)	40.00 (33.00–49.00)	<0.001
Sex, *n* (%)				<0.001
Male	69 (61.1)	56 (87.5)	48 (73.8)	
Female	44 (38.9)	8 (12.5)	17 (26.2)	
HBV DNA category, *n* (%)				<0.001
<100	30 (26.5)	62 (96.9)	62 (95.4)	
≥100	83 (73.5)	2 (3.1)	3 (4.6)	
Body weight, kg, median (IQR)	64.50 (55.00–70.50)	66.00 (58.00–70.78)	62.50 (55.00–68.00)	0.150
ALT, U/L, median (IQR)	29.90 (21.10–50.50)	22.80 (15.60–30.95)	28.80 (22.50–35.94)	0.002
Serum creatinine, μmol/L, median (IQR)	68.20 (55.60–80.60)	78.50 (67.10–86.45)	76.30 (67.90–86.80)	<0.001
Uric acid, μmol/L, median (IQR)	335.90 (280.80–398.10)	366.30 (303.00–410.15)	319.50 (267.70–420.80)	0.284
HBsAg, IU/mL, median (IQR)	1,413.87 (173.49–3,540.00)	623.26 (195.17–1,704.33)	1,770.74 (603.40–3,537.20)	0.002
Urinary A1M, mg/L, median (IQR)	3.80 (2.00–7.40)	11.35 (5.23–22.92)	15.80 (9.10–21.50)	<0.001
Urinary B2M, mg/L, median (IQR)	0.17 (0.09–0.30)	0.27 (0.14–0.83)	0.48 (0.30–1.97)	<0.001
U-mAlb, mg/L, median (IQR)	5.60 (2.00–14.60)	22.55 (11.65–48.23)	19.90 (11.80–34.20)	<0.001
eGFR, mL/min/1.73 m^2^, median (IQR)	112.98 (102.47–119.36)	104.79 (92.08–112.39)	104.47 (91.24–116.58)	<0.001
Hypertension, *n* (%)	4 (3.5)	7 (10.9)	2 (3.1)	0.104
Diabetes mellitus, *n* (%)	1 (0.9)	2 (3.1)	2 (3.1)	0.528
Hyperuricemia, *n* (%)	6 (5.3)	5 (7.8)	2 (3.1)	0.467
Fatty liver disease, *n* (%)	36 (31.9)	13 (20.3)	9 (13.8)	0.019
Compensated cirrhosis, *n* (%)	2 (1.8)	5 (7.8)	3 (4.6)	0.145

Continuous variables are presented as median [interquartile range (IQR)], with IQR representing the 25th–75th percentiles. Categorical variables are presented as *n* (%), with percentages calculated within the corresponding antiviral exposure group. Overall *P* values represent comparisons across the three groups and were calculated using the Kruskal–Wallis test for continuous variables and the chi-square test or Fisher's exact test for categorical variables, as appropriate. *Post hoc* pairwise comparisons with Bonferroni adjustment are presented separately in Table 2 for continuous variables and Table 3 for categorical variables. HBV DNA was categorized as <100 or ≥100 IU/mL. ALT, alanine aminotransferase; A1M, *α*1-microglobulin; B2M, *β*2-microglobulin; eGFR, estimated glomerular filtration rate; ETV, entecavir; HBsAg, hepatitis B surface antigen; NA, nucleos(t)ide analogue; TDF, tenofovir disoproxil fumarate; U-mAlb, urinary microalbumin. Urinary A1M, B2M, and U-mAlb are reported as unnormalized spot-urine concentrations (mg/L); paired urinary creatinine was unavailable, so creatinine-normalized ratios were not calculated.

Bonferroni-adjusted pairwise analyses showed that participants in the ETV-treated group were older than those in both the NA-untreated and TDF-treated groups, whereas age was comparable between the NA-untreated and TDF-treated groups. The proportion of male participants was higher in the ETV-treated group than in the NA-untreated group, while the other pairwise comparisons for sex were not statistically significant. HBV DNA ≥100 IU/mL was more frequent in the NA-untreated group than in either treated group, with no significant difference between the ETV-treated and TDF-treated groups. This marked separation in HBV DNA status indicates that the NA-untreated group represented a substantially different virological state at the index assessment. The NA-untreated group is therefore used as a real-world reference group rather than as a counterfactual untreated control for estimating antiviral drug effects.

ALT levels were lower in the ETV-treated group than in both the NA-untreated and TDF-treated groups. Serum creatinine levels were higher and eGFR values were lower in both treated groups than in the NA-untreated group, whereas neither measure differed significantly between the ETV-treated and TDF-treated groups. Urinary A1M, B2M, and U-mAlb levels were higher in both treated groups than in the NA-untreated group. Urinary B2M was also higher in the TDF-treated group than in the ETV-treated group, whereas urinary A1M and U-mAlb did not differ significantly between the two treated groups. HBsAg levels were lower in the ETV-treated group than in the TDF-treated group, while the other pairwise comparisons were not statistically significant. Fatty liver disease was more frequent in the NA-untreated group than in the TDF-treated group, with no significant differences in the other pairwise comparisons. Detailed results of the Bonferroni-adjusted pairwise comparisons are presented in Tables 2, 3. These urinary biomarker comparisons are based on unnormalized spot-urine concentrations and therefore remain susceptible to variation in urine concentration.

**Table 2:** *Post hoc* pairwise comparisons of continuous variables among NA-untreated, ETV-treated, and TDF-treated groups.

Variable	Overall *P* value	NA-untreated vs. ETV-treated (adjusted *P*)	NA-untreated vs. TDF-treated (adjusted *P*)	ETV-treated vs. TDF-treated (adjusted *P*)
Age	<0.001	<0.001	1.0000	0.0052
ALT	0.0017	0.0027	0.7851	0.0204
Serum creatinine	0.0004	0.0028	0.0045	1.0000
HBsAg	0.0022	0.0949	0.4759	0.0006
Urinary A1M	<0.001	<0.001	<0.001	0.2684
Urinary B2M	<0.001	0.0003	<0.001	0.0074
U-mAlb	<0.001	<0.001	<0.001	0.9641
eGFR	0.0008	0.0026	0.0013	1.0000

Overall comparisons were performed using the Kruskal–Wallis test. Pairwise comparisons were performed using Mann–Whitney *U* tests, and *P* values were adjusted for three pairwise comparisons using the Bonferroni method. An adjusted *P* value <0.05 was considered statistically significant. ALT, alanine aminotransferase; A1M, *α*1-microglobulin; B2M, *β*2-microglobulin; eGFR, estimated glomerular filtration rate; ETV, entecavir; HBsAg, hepatitis B surface antigen; NA, nucleos(t)ide analogue; TDF, tenofovir disoproxil fumarate; U-mAlb, urinary microalbumin.

**Table 3:** *Post hoc* pairwise comparisons of categorical variables among NA-untreated, ETV-treated, and TDF-treated groups.

Variable	Overall *P* value	NA-untreated vs. ETV-treated (adjusted *P*)	NA-untreated vs. TDF-treated (adjusted *P*)	ETV-treated vs. TDF-treated (adjusted *P*)
HBV DNA ≥100 IU/mL	<0.001	<0.001	<0.001	1.0000
Male	0.0008	0.0006	0.2507	0.1494
Fatty liver disease	0.0185	0.2972	0.0233	0.9867
Reduced eGFR (<90 mL/min/1.73 m^2^)	<0.001	0.0025	0.0012	1.0000
Urinary A1M ≥ 12.0 mg/L	<0.001	<0.001	<0.001	0.0108
Urinary B2M > 0.30 mg/L	<0.001	0.0063	<0.001	0.0048
U-mAlb ≥15.0 mg/L	<0.001	<0.001	<0.001	0.8362

Overall comparisons were performed using the Pearson chi-square test or Fisher–Freeman–Halton exact test, as appropriate. *Post hoc* pairwise comparisons were performed using pairwise Pearson chi-square tests or Fisher's exact tests, as appropriate, with Bonferroni adjustment for three pairwise comparisons. Only one category is displayed for each binary variable because the complementary category yields the same overall and pairwise *P* values. An adjusted *P* value <0.05 was considered statistically significant. The urinary biomarker categories are based on spot-urine concentration thresholds (mg/L) and are not creatinine-normalized.

### 3.2 Urinary tubular biomarker patterns and glomerular-function findings by antiviral exposure group

The most distinct direct ETV-vs.-TDF differences were observed in the urinary low-molecular-weight biomarkers. The proportion of participants with urinary A1M ≥ 12.0 mg/L was 13.3%, 42.2%, and 67.7% in the NA-untreated, ETV-treated, and TDF-treated groups, respectively; the corresponding proportions with urinary B2M > 0.30 mg/L were 25.7%, 48.4%, and 75.4%. Overall differences were significant for both concentration-threshold outcomes (both *P* < 0.001). After Bonferroni adjustment, A1M and B2M threshold exceedance were both more frequent in TDF-treated than ETV-treated participants (adjusted *P* = 0.0108 and *P* = 0.0048, respectively), and both treated groups had higher proportions than the NA-untreated group (Table 3). Consistent with this pattern, continuous urinary B2M concentration was higher in TDF than ETV (adjusted *P* = 0.0074), whereas continuous urinary A1M concentration did not differ between the two treated groups (adjusted *P* = 0.2684) (Table 2). U-mAlb ≥15.0 mg/L was observed in 23.0%, 73.4%, and 64.6% of participants, respectively, but did not differ between ETV and TDF after adjustment (Table 3). These urinary findings are based on unnormalized spot-urine concentrations and should be interpreted as concentration-based biomarker patterns rather than standardized measures of urinary excretion or definitive evidence of tubular injury. Urinary B2M requires additional pre-analytical caution because participant-level urine pH, processing interval, storage conditions, and sample-stability information were unavailable. Because a complete proximal tubular dysfunction panel was unavailable, these A1M/B2M differences were not classified as Fanconi syndrome or definite TDF-induced proximal tubular toxicity.

By contrast, conventional glomerular-function measures did not distinguish the two treated groups. Serum creatinine did not differ between ETV and TDF in the direct pairwise comparison (Bonferroni-adjusted *P* = 1.0000) (Table 2). Continuous eGFR differed across the three exposure groups (Kruskal–Wallis *P* = 0.0008), with median (IQR) values of 112.98 (102.47–119.36), 104.79 (92.08–112.39), and 104.47 (91.24–116.58) mL/min/1.73 m^2^ in the NA-untreated, ETV-treated, and TDF-treated groups, respectively. eGFR was lower in both treated groups than in the NA-untreated group after Bonferroni adjustment, but did not differ between ETV and TDF (adjusted *P* = 1.0000) (Tables 1, 2). The secondary eGFR <90 mL/min/1.73 m² screening outcome was observed in 5.3%, 21.9%, and 23.1% of the three groups, respectively; it likewise did not differ between ETV and TDF (adjusted *P* = 1.0000) (Tables 3 and 4). An eGFR <60 mL/min/1.73 m^2^ was uncommon (0, 1, and 2 participants, respectively; overall *P* = 0.150). Thus, the present dataset identifies a more prominent urinary A1M/B2M pattern in TDF-treated participants without a parallel ETV-vs.-TDF difference in serum creatinine or eGFR.

**Table 4:** Prevalence of reduced eGFR and urinary biomarker values above institutional concentration thresholds by antiviral exposure group.

Indicator, *n* (%)	NA-untreated (*n* = 113)	ETV-treated (*n* = 64)	TDF-treated (*n* = 65)	Total (*n* = 242)	Overall *P* value
Reduced eGFR (<90 mL/min/1.73 m^2^)	6 (5.3)	14 (21.9)	15 (23.1)	35 (14.5)	<0.001
eGFR <60 mL/min/1.73 m^2^	0 (0.0)	1 (1.6)	2 (3.1)	3 (1.2)	0.150
Urinary A1M ≥ 12.0 mg/L	15 (13.3)	27 (42.2)	44 (67.7)	86 (35.5)	<0.001
Urinary B2M > 0.30 mg/L	29 (25.7)	31 (48.4)	49 (75.4)	109 (45.0)	<0.001
U-mAlb ≥15.0 mg/L	26 (23.0)	47 (73.4)	42 (64.6)	115 (47.5)	<0.001

Values are presented as *n* (%), with percentages calculated using the total number of participants in the corresponding antiviral exposure group as the denominator. Overall comparisons were performed using the chi-square test or the Fisher–Freeman–Halton exact test, as appropriate. *Post hoc* pairwise comparisons with Bonferroni adjustment were performed only for variables with a statistically significant overall comparison and are presented in Table 3. The urinary biomarker categories correspond to the institutional concentration thresholds specified in the Methods section. A1M, *α*1-microglobulin; B2M, *β*2-microglobulin; eGFR, estimated glomerular filtration rate; ETV, entecavir; NA, nucleos(t)ide analogue; TDF, tenofovir disoproxil fumarate; U-mAlb, urinary microalbumin. Paired urinary creatinine was unavailable, and no A1M/Cr, B2M/Cr, or albumin-to-creatinine ratios were calculated. Accordingly, U-mAlb ≥15.0 mg/L denotes only concentration-threshold exceedance and not a standardized albuminuria category.

The distributions of serum creatinine, uric acid, eGFR, urinary A1M, urinary B2M, and U-mAlb across the three antiviral exposure groups are shown in Supplementary Figures S1–S6, respectively.

### 3.3 Exploratory analysis of factors associated with the eGFR <90 mL/min/1.73 m^2^ screening outcome

As a secondary exploratory analysis, binary logistic regression was performed for the categorical screening outcome of eGFR <90 mL/min/1.73 m^2^. Model 1 was unadjusted. In view of the limited number of outcome events (35/242), the adjusted analysis was restricted to a parsimonious Model 2 including antiviral exposure group, age, and sex (Table 5); the previously reported more highly parameterized Model 3 was removed to reduce overfitting risk. Age and sex were treated only as adjustment terms and their coefficients were not interpreted as independent biological associations because both variables are components of the 2009 CKD-EPI creatinine equation used to calculate eGFR.

**Table 5:** Parsimonious exploratory logistic regression models for the eGFR <90 mL/min/1.73 m^2^ screening outcome.

Variable	Model 1		Model 2	
	OR (95% CI)	*P* value	OR (95% CI)	*P* value
ETV-treated vs. NA-untreated	4.993 (1.812–13.759)	0.002	4.024 (1.269–12.753)	0.018
TDF-treated vs. NA-untreated	5.350 (1.959–14.609)	0.001	5.910 (2.009–17.385)	0.001
Age, per year	—	—	1.092 (1.046–1.139)	<0.001
Male vs. female	—	—	0.323 (0.130–0.802)	0.015

The dependent variable was the secondary eGFR <90 mL/min/1.73 m^2^ screening outcome. Results are presented as odds ratios (ORs) with 95% confidence intervals (CIs). Only 35 participants met this outcome; therefore, the adjusted model was intentionally parsimonious and the estimates should be interpreted as exploratory and imprecise.

The NA-untreated group and female sex were used as the reference categories.

Model 1 was unadjusted. Model 2 included antiviral exposure group, age, and sex. No composite comorbidity covariate was used in the final regression because the individual conditions were clinically heterogeneous; inclusion of all five comorbidities separately was not considered stable given the sparse prevalence of several conditions and the limited number of outcome events. Because age and sex are components of the 2009 CKD-EPI creatinine equation used to calculate eGFR, their coefficients are reported only for transparency and are not interpreted as independent biological risk or protective effects.

In Model 1, ETV and TDF exposure were associated with higher odds of the eGFR <90 mL/min/1.73 m^2^ screening outcome compared with the NA-untreated group. In the parsimonious Model 2, the OR was 4.024 for ETV-treated vs. NA-untreated participants (95% CI: 1.269–12.753; *P* = 0.018) and 5.910 for TDF-treated vs. NA-untreated participants (95% CI: 2.009–17.385; *P* = 0.001). These confidence intervals remained wide, indicating limited precision; accordingly, the estimates are interpreted only as exploratory cross-sectional associations and not as stable effect sizes or causal drug-effect estimates. Age and sex were included only as adjustment covariates and their coefficients were not interpreted biologically.

### 3.4 Sensitivity analyses

Two sensitivity analyses were conducted. First, when an eGFR <60 mL/min/1.73 m^2^ was used as a stricter renal outcome, only three events were identified in the entire study population: none in the NA-untreated group, one in the ETV-treated group, and two in the TDF-treated group. Because of the limited number of events, adjusted logistic regression was not performed for this outcome. Second, in an exploratory analysis restricted to ETV- and TDF-treated participants, TDF exposure was not associated with the eGFR <90 mL/min/1.73 m^2^ screening outcome compared with ETV after adjustment for age, sex, body weight, and any documented comorbidity (adjusted OR: 1.039; 95% CI: 0.378–2.857; *P* = 0.940). Because the composite comorbidity variable does not capture heterogeneity among individual conditions and the treated-only event count was limited, this estimate is interpreted as supportive and imprecise rather than as evidence of equivalence or robust confounding control (Table 6). The previously reported sensitivity analysis excluding all participants with documented comorbidities was not retained because of sparse events and very wide confidence intervals.

**Table 6:** Sensitivity analyses for reduced eGFR.

Sensitivity analysis	Comparison	Adjusted OR (95% CI)	*P* value	Additional information
Stricter renal outcome: eGFR <60 mL/min/1.73 m^2^	Descriptive analysis only	—	—	NA-untreated: 0/113; ETV-treated: 1/64; TDF-treated: 2/65
Analysis restricted to treated participants	TDF-treated vs. ETV-treated	1.039 (0.378–2.857)	0.940	Adjusted for age, sex, body weight, and any documented comorbidity

Reduced eGFR was defined as eGFR <90 mL/min/1.73 m^2^ for the treated-only model. The treated-only analysis was adjusted for age, sex, body weight, and the presence of any documented comorbidity. Any documented comorbidity was defined as the presence of at least one of the following conditions: hypertension, diabetes mellitus, hyperuricemia, fatty liver disease, or compensated cirrhosis. Because this composite combines clinically heterogeneous conditions and the number of outcome events was limited, the treated-only estimate should be interpreted as exploratory. Adjusted logistic regression for the eGFR <60 mL/min/1.73 m^2^ outcome was not performed because only three events were observed.

### 3.5 Group-specific spearman correlation analysis of laboratory indicators

**Figure 2:**
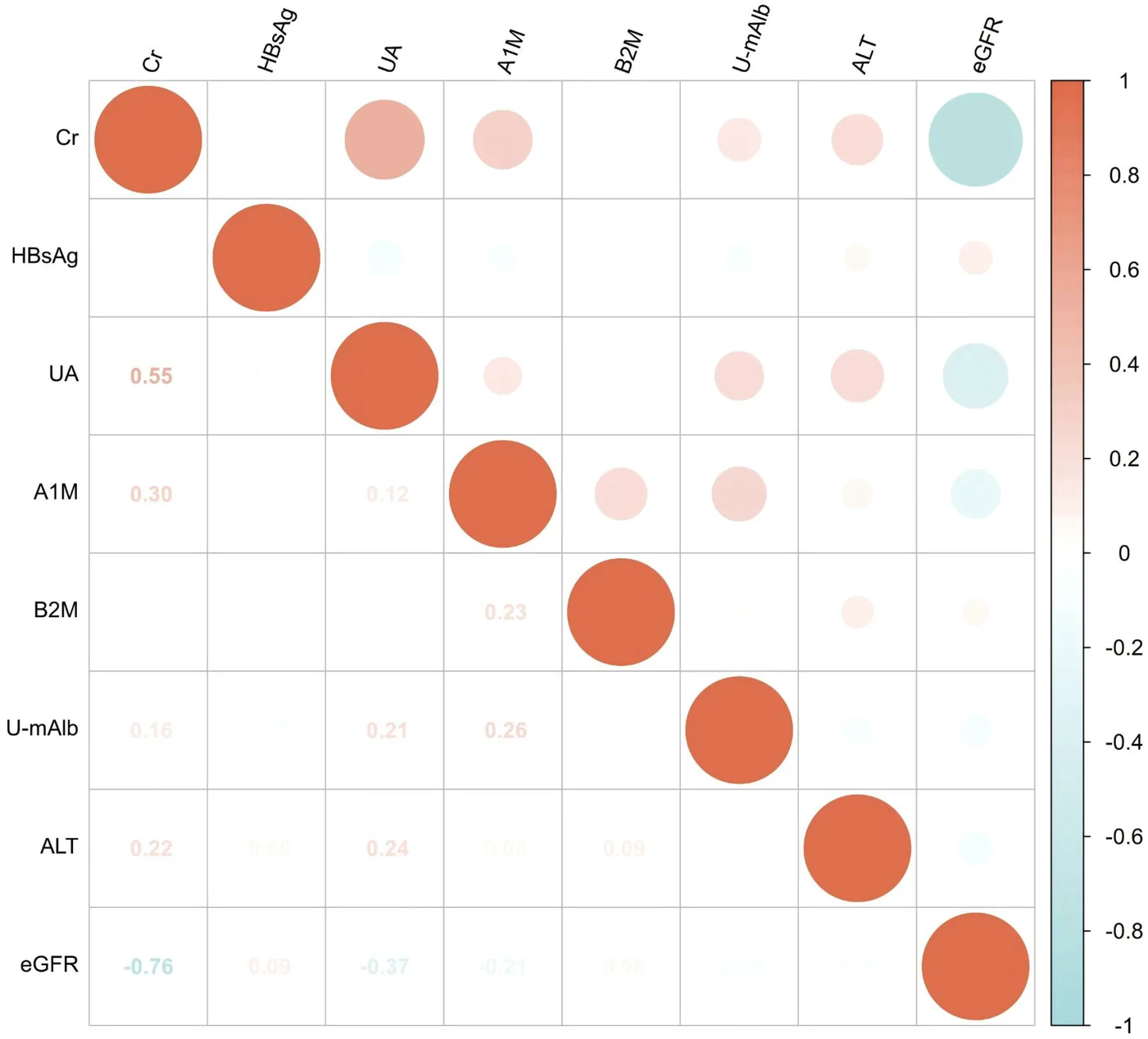
Spearman correlation matrix of laboratory indicators in the NA-untreated group. The lower triangle displays Spearman's correlation coefficients (*ρ*), whereas the upper triangle uses circles to represent the direction and magnitude of the correlations. Larger circles and greater color intensity indicate stronger correlations; warm colors indicate positive correlations and cool colors indicate negative correlations. Cr, serum creatinine; HBsAg, hepatitis B surface antigen; UA, uric acid; A1M, *α*1-microglobulin; B2M, *β*2-microglobulin; U-mAlb, urinary microalbumin; ALT, alanine aminotransferase; eGFR, estimated glomerular filtration rate; ETV, entecavir; NA, nucleos(t)ide analogue; TDF, tenofovir disoproxil fumarate.

**Figure 3:**
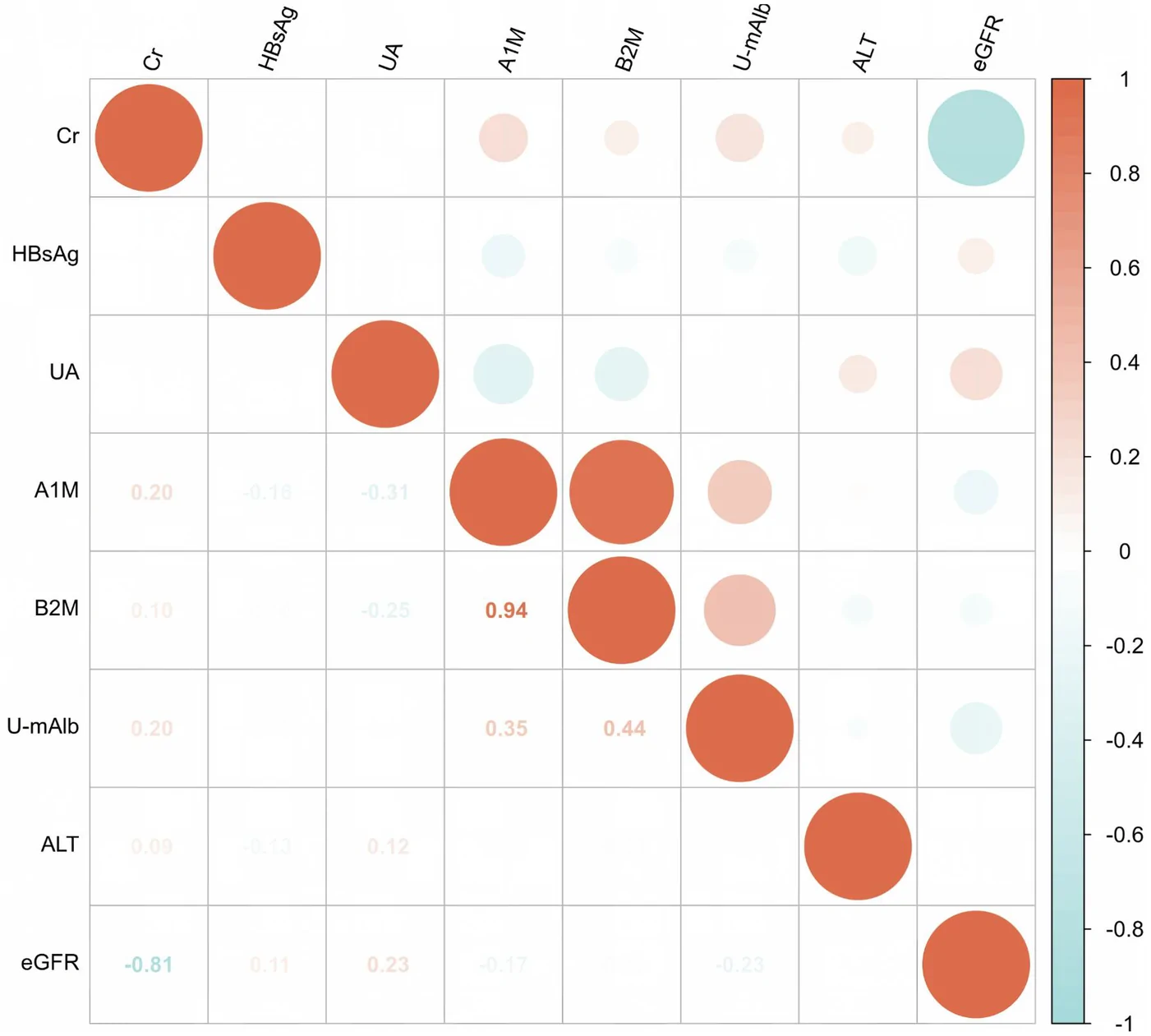
Spearman correlation matrix of laboratory indicators in the ETV-treated group. The lower triangle displays Spearman's correlation coefficients (*ρ*), whereas the upper triangle uses circles to represent the direction and magnitude of the correlations. Larger circles and greater color intensity indicate stronger correlations; warm colors indicate positive correlations and cool colors indicate negative correlations. Cr, serum creatinine; HBsAg, hepatitis B surface antigen; UA, uric acid; A1M, *α*1-microglobulin; B2M, *β*2-microglobulin; U-mAlb, urinary microalbumin; ALT, alanine aminotransferase; eGFR, estimated glomerular filtration rate; ETV, entecavir; NA, nucleos(t)ide analogue; TDF, tenofovir disoproxil fumarate.

**Figure 4:**
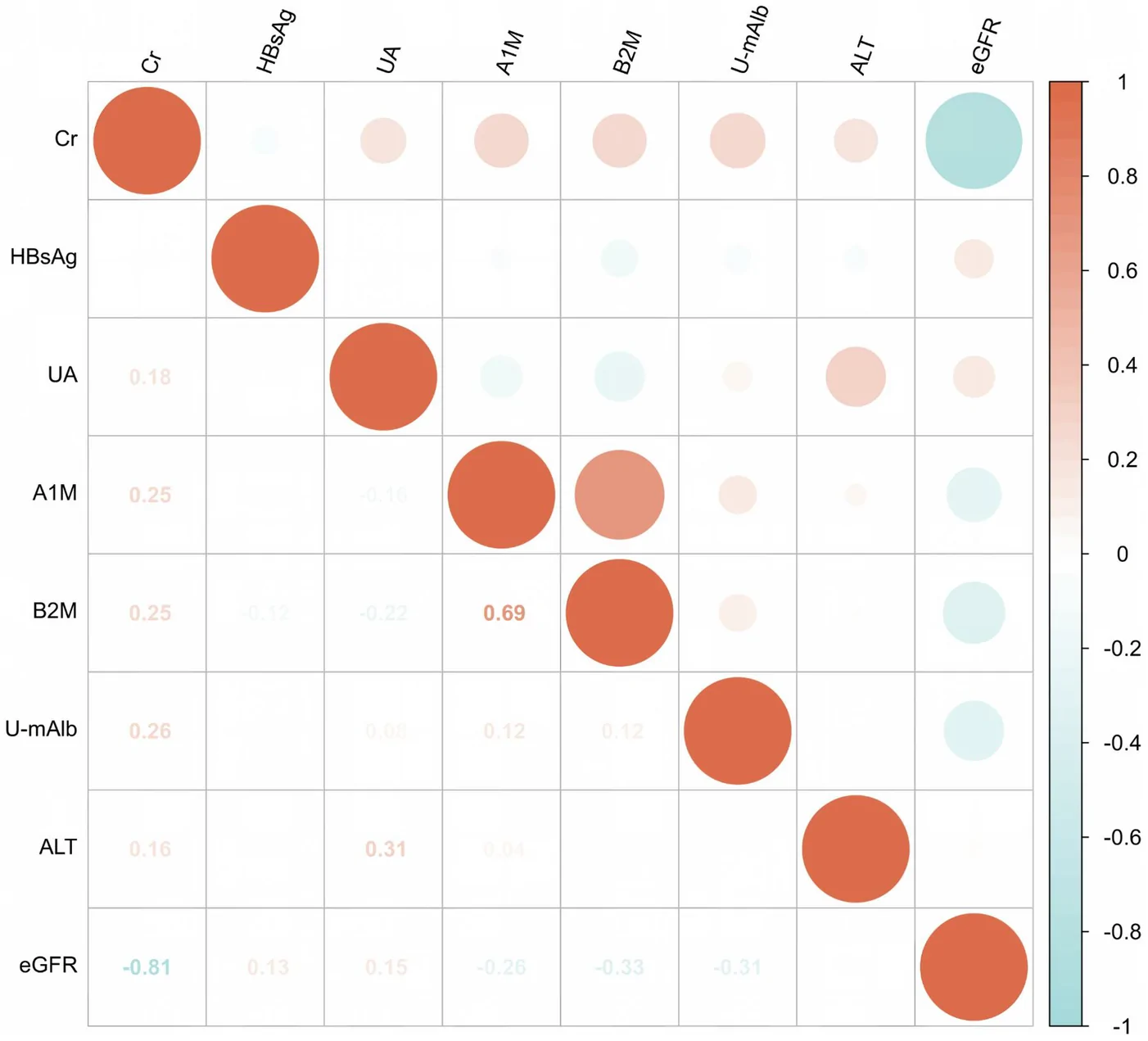
Spearman correlation matrix of laboratory indicators in the TDF-treated group. The lower triangle displays Spearman's correlation coefficients (*ρ*), whereas the upper triangle uses circles to represent the direction and magnitude of the correlations. Larger circles and greater color intensity indicate stronger correlations; warm colors indicate positive correlations and cool colors indicate negative correlations. Cr, serum creatinine; HBsAg, hepatitis B surface antigen; UA, uric acid; A1M, *α*1-microglobulin; B2M, *β*2-microglobulin; U-mAlb, urinary microalbumin; ALT, alanine aminotransferase; eGFR, estimated glomerular filtration rate; ETV, entecavir; NA, nucleos(t)ide analogue; TDF, tenofovir disoproxil fumarate.

Spearman rank correlation analyses were conducted separately in the NA-untreated, ETV-treated, and TDF-treated groups to examine the relationships among serum creatinine, HBsAg, uric acid, urinary A1M, urinary B2M, U-mAlb, ALT, and eGFR (Figures 2–4). The analyses were exploratory, and the correlation coefficients were used to describe the direction and magnitude of the observed associations within each exposure group. Because the analysis included variables that are mathematically related, particularly serum creatinine and eGFR, those coefficients were reported only for completeness and were not used to support biological inference or the principal conclusions of the study.

In the NA-untreated group, serum creatinine was strongly inversely correlated with eGFR (Spearman's *ρ* = −0.76) and moderately positively correlated with uric acid (*ρ* = 0.55). Uric acid was inversely correlated with eGFR (*ρ* = −0.37). Positive correlations were also observed between serum creatinine and urinary A1M (*ρ* = 0.30), between urinary A1M and U-mAlb (*ρ* = 0.26), and between urinary A1M and B2M (*ρ* = 0.23). Most correlations involving HBsAg or ALT were weak in magnitude (Figure 2).

In the ETV-treated group, urinary A1M and B2M showed a very strong positive correlation (*ρ* = 0.94). Urinary B2M was positively correlated with U-mAlb (*ρ* = 0.44), and urinary A1M was positively correlated with U-mAlb (*ρ* = 0.35). Serum creatinine was strongly inversely correlated with eGFR (*ρ* = −0.81). Uric acid was inversely correlated with urinary A1M (*ρ* = −0.31) and B2M (*ρ* = −0.25), whereas the remaining correlations involving ALT and HBsAg were generally weak (Figure 3). This very high coefficient is retained as a descriptive observation rather than interpreted as evidence of an ETV-specific biological effect. Because A1M and B2M were measured as unnormalized spot-urine concentrations, a shared effect of urine concentration/dilution cannot be excluded, and B2M-specific pre-analytical factors provide an additional source of uncertainty.

In the TDF-treated group, serum creatinine was strongly inversely correlated with eGFR (*ρ* = −0.81). Urinary A1M and B2M were positively correlated (*ρ* = 0.69). eGFR showed inverse correlations with urinary B2M (*ρ* = −0.33), U-mAlb (*ρ* = −0.31), and A1M (*ρ* = −0.26). Serum creatinine was positively correlated with urinary A1M (*ρ* = 0.25), B2M (*ρ* = 0.25), and U-mAlb (*ρ* = 0.26). Correlations of ALT and HBsAg with renal function and urinary biomarkers were weak in magnitude (Figure 4).

Across the three groups, the inverse correlations between serum creatinine and eGFR were expected by construction because serum creatinine was used to calculate eGFR; these coefficients are therefore reported only for completeness and were not regarded as biologically informative findings. Positive correlations among the urinary biomarkers were observed within the exposure groups, including the A1M–B2M correlation in the ETV-treated group. Because no formal statistical comparisons of correlation coefficients were performed across the exposure groups, numerical differences in *ρ* values were not interpreted as true between-group differences in correlation strength. The terms “stronger” and “weaker” are therefore not used to infer cross-group differences from these descriptive matrices. In particular, although the A1M–B2M correlation of *ρ* = 0.94 in the ETV-treated group is biologically plausible because both low-molecular-weight proteins are filtered and predominantly reabsorbed by proximal tubular cells, the present data cannot separate shared tubular handling from shared urine concentration or other common technical influences. We therefore do not interpret the magnitude of this coefficient as an ETV-specific mechanistic signal.

## 4 Discussion

In this single-center retrospective cross-sectional study, participants receiving ETV or TDF showed lower median eGFR and more frequent urinary biomarker threshold exceedance than NA-untreated participants. The clearest distinction between the two treated groups, however, was in urinary A1M and B2M: concentration-threshold exceedance was more frequent with TDF, whereas serum creatinine, continuous eGFR, and the eGFR <90 mL/min/1.73 m^2^ screening outcome did not differ between ETV and TDF. These findings suggest that tubular biomarker patterns and conventional glomerular-function measures may capture different aspects of renal involvement. Because renal parameters were assessed at a single index time point, the observed differences should be interpreted as cross-sectional associations rather than treatment-induced renal injury or renal decline.

Previous studies have reported heterogeneous renal safety findings across first-line nucleos(t)ide analogues, depending on baseline kidney function, treatment duration, and the renal outcome assessed. A large longitudinal cohort reported a greater risk of chronic kidney disease progression with TDF than with no antiviral treatment (23), whereas a network meta-analysis suggested that differences between ETV and TDF were generally small and became more apparent with longer exposure (9). Long-term TAF trials have also shown favorable renal and bone safety profiles (24). Our findings fit this broader literature but add a different real-world perspective: within one cohort, urinary low-molecular-weight biomarker differences were more apparent than glomerular-function differences between ETV and TDF. Thus, the contribution of the present study is comparative rather than mechanistic, and the data do not support a generalized conclusion that TDF caused greater overall renal impairment than ETV.

The A1M/B2M pattern observed in the TDF-treated group is biologically plausible. Tenofovir can accumulate within proximal tubular cells and interfere with mitochondrial and transport-dependent functions (25). A1M and B2M are low-molecular-weight proteins that are filtered by the glomerulus and largely reabsorbed by proximal tubular cells; increased urinary concentrations may therefore accompany impaired tubular reabsorptive handling (14, 15). Urinary B2M has also been evaluated as a marker of tubular dysfunction in TDF-treated patients with CHB (19). Nevertheless, the present measurements were unnormalized spot-urine concentrations, and a complete proximal tubular dysfunction panel—including serum phosphate, phosphate wasting, glycosuria, and bicarbonate—was unavailable. In addition, urinary B2M is pH-sensitive, and participant-level information on urine pH and specimen handling was not available (22). Accordingly, the A1M/B2M findings should be regarded as a supportive tubular biomarker pattern, not as a diagnosis of Fanconi syndrome or definite TDF-induced proximal tubular toxicity.

The eGFR findings require a different caution. Antiviral treatment selection in routine practice is not random, and patients perceived to have greater renal or bone vulnerability may be more likely to receive ETV or TAF and less likely to receive TDF, creating confounding by indication (20, 26). In our cohort, the ETV-treated group was older, and the exposure groups also differed in sex distribution, HBV DNA status, ALT, and fatty liver disease at the index assessment. The pronounced HBV DNA difference between treated and untreated patients reflects different virological states, but index HBV DNA in treated patients was measured after antiviral exposure and therefore cannot simply be treated as a pretreatment confounder. Pretreatment renal function, precise treatment duration, cumulative exposure, prescribing rationale, dose adjustment, adherence, and the severity or control of comorbidities were unavailable. These factors limit direct comparability and cannot be fully resolved by multivariable adjustment. Age and sex are also components of the 2009 CKD-EPI equation, so their coefficients in the eGFR-threshold model should not be interpreted as independent biological effects (17). The treated-only sensitivity analysis likewise showed no significant difference in reduced eGFR between TDF and ETV, reinforcing that the present data do not demonstrate worse glomerular function with TDF.

The exploratory correlation analysis was intended to describe within-group relationships rather than test treatment effects. The strong inverse association between serum creatinine and eGFR is mathematically expected because serum creatinine is used in the 2009 CKD-EPI equation (17) and therefore provides little independent biological information. Positive correlations among A1M, B2M, and U-mAlb may reflect partially overlapping renal handling, but no individual urinary biomarker is sufficiently specific to establish drug-induced nephrotoxicity (20). Population-based studies also suggest that urinary tubular biomarkers may provide complementary information beyond conventional renal measures (27, 28). The very high A1M–B2M correlation in the ETV-treated group (*ρ* = 0.94) is biologically plausible because the two proteins share proximal tubular handling, yet a common urine concentration effect and B2M-specific pre-analytical factors cannot be excluded. Because no formal between-group tests of correlation coefficients were performed, numerical differences in correlation strength across groups were not interpreted as true between-group differences.

From a clinical perspective, these findings favor individualized renal assessment rather than automatic changes in antiviral therapy. Current HBV guidance recommends considering baseline kidney function, age, comorbidities, and renal or bone risk factors when selecting and monitoring therapy (20). Long-term phase 3 data support the renal safety of TAF (29), and switching studies in virologically suppressed patients with renal or hepatic impairment have reported favorable outcomes after transition to TAF (30, 31). However, the present study did not include a TAF-treated group or a longitudinal switching cohort and therefore cannot determine whether switching therapy would reverse the observed urinary biomarker pattern or improve future renal outcomes. Such implications should be based on external longitudinal evidence rather than on the present cross-sectional data.

Several limitations should be considered when interpreting the glomerular-function analyses. Pretreatment renal measurements and serial follow-up were unavailable, so temporal sequence, within-person renal trajectories, reversibility, and duration-response relationships could not be assessed. Continuous eGFR therefore provides the principal description of glomerular function, while eGFR <90 mL/min/1.73 m^2^ is retained only as a secondary screening outcome; a single value below 90 does not establish chronic kidney disease (12). Only 35 participants met this screening threshold, resulting in wide confidence intervals and limited precision in the exploratory regression (32). The model was therefore kept parsimonious, and the unstable sensitivity analysis excluding all participants with comorbidities was not retained. Creatinine-based eGFR also remains susceptible to non-GFR determinants such as muscle mass, and cystatin C or measured GFR was unavailable for independent confirmation.

Additional limitations relate to exposure and urinary biomarker measurement. The ≥12-month treatment requirement was an eligibility threshold rather than a measure of cumulative exposure; exact treatment duration, antiviral dose, dose adjustment, and formal adherence were unavailable. The five comorbidities were reported separately because they represent heterogeneous renal risk states, but their severity, duration, treatment, and control were incompletely characterized, leaving residual confounding. Urinary A1M, B2M, and U-mAlb were measured as spot concentrations without urinary creatinine normalization, so urine concentration or dilution may have contributed to between-group differences; U-mAlb concentration should not be interpreted as a standardized albuminuria category. B2M also has the pre-analytical limitation noted above. Finally, the single-center design, modest sample size, different clinical and virological states across exposure groups, and incomplete pretreatment liver disease staging restrict generalizability and causal interpretation. The study should therefore be viewed as a real-world cross-sectional comparison of renal biomarker patterns after ≥12 months of therapy, not as evidence of dynamic monitoring, long-term renal safety, or treatment-induced nephrotoxicity.

## 5 Conclusion

In conclusion, the principal ETV–TDF distinction in this retrospective cross-sectional study was a urinary tubular biomarker pattern rather than a difference in glomerular function. TDF-treated participants had more frequent A1M and B2M concentration-threshold exceedance than ETV-treated participants, while serum creatinine, continuous eGFR, and the secondary eGFR <90 mL/min/1.73 m^2^ screening outcome did not differ between the two treated groups. The present findings therefore should not be summarized as generalized renal impairment with TDF. Moreover, the A1M/B2M pattern does not establish definite TDF-induced tubular injury because the urinary measurements were not normalized to creatinine, B2M-specific pre-analytical information was incomplete, and a full proximal tubular dysfunction panel was unavailable. Pretreatment renal function, serial measurements, exact treatment duration, cumulative exposure, dose-adjustment history, and adherence were also unavailable. These limitations restrict the findings to cross-sectional comparative biomarker patterns. Prospective longitudinal studies incorporating baseline and serial glomerular and tubular measurements, creatinine-normalized urinary biomarkers, standardized B2M handling, and detailed antiviral exposure are needed to determine the temporal and clinical significance of this pattern. Specifically, the study did not include a complete diagnostic assessment for proximal tubular dysfunction, including serum phosphate, phosphate wasting, glycosuria, and bicarbonate; consequently, neither Fanconi syndrome nor definite TDF-induced proximal tubular toxicity was established. For clarity, the ≥12-month treatment criterion was an exposure eligibility threshold only; the present study is best described as a cross-sectional comparison of renal biomarkers at a single index assessment after ≥12 months of therapy, not as long-term renal safety monitoring or an analysis of renal trajectories. No causal attribution of these between-group differences to ETV or TDF is made from the present cross-sectional data. Accordingly, the contribution of this study lies in the simultaneous real-world comparative characterization of untreated, ETV-treated, and TDF-treated patients across both tubular biomarker and glomerular-function domains, rather than in proposing a novel mechanism of TDF nephrotoxicity.

## Data Availability

The original contributions presented in the study are included in the article/Supplementary Material, further inquiries can be directed to the corresponding author.
